# Post-traumatic pseudoaneurysm of the anterior tibial artery: therapeutic challenge

**DOI:** 10.1590/1677-5449.202401202

**Published:** 2025-03-14

**Authors:** Poliana Mazuchini Belai, Zaira Cristina Barbosa Assis, Thiago Felipe de Moraes Vieira, Gislaine dos Santos Rodrigues Vieira, Henrique Alves de Almeida, Thiago Vaz Lopes, João da Silva, Vinicius Tadeu Ramos da Silva Grillo

**Affiliations:** 1 Centro Universitário São Lucas – UNISL-Afya, Porto Velho, RO, Brasil.; 2 Instituto Vascular e Endovascular de Rondônia, Porto Velho, RO, Brasil.

**Keywords:** pseudoaneurysm, anterior tibial artery, vascular injuries

## Abstract

Subacute traumatic arterial pseudoaneurysms involving the infragenicular arteries are rarely discussed in the literature because they tend to be asymptomatic or manifest clinically subtle symptoms even at late stages. We describe the case of a 25-year-old patient with a perforation injury to the lower limb from a shard of glass, who, after two weeks, developed pain and swelling in the limb, which suggested deep vein thrombosis. Venous Doppler ultrasound ruled out thrombosis but revealed heterogeneous echogenicity in the anterior wall of the middle third of the anterior tibial artery with internal flow, suggesting a pseudoaneurysm. We opted for conventional surgical treatment and, after proximal and distal control of the artery, drainage of the local hematoma and primary suturing of the lesion were performed. Pseudoaneurysm of the infrapatellar arteries, although rare, is a challenging clinical entity that requires early diagnosis.

## INTRODUCTION

Pseudoaneurysms can originate in any artery in the human body. In the lower limbs, the popliteal artery is most commonly affected, followed by the superficial femoral artery and the anterior tibial artery (ATA). The ATA is the blood vessel most frequently associated with infrapatellar pseudoaneurysm, although its incidence is relatively rare.^1-3^

Pseudoaneurysms of the ATA are uncommon events that arise due to various iatrogenic causes or to blunt or penetrating trauma. Despite their rarity, these injuries are associated with significant morbidity, often posing a threat to limb integrity.^1,2,4,5^

The interval between the initial injury and the appearance of the pseudoaneurysm can vary from hours to years, depending on the location, size, and clinical manifestations. A high index of clinical suspicion is essential for accurate diagnosis of vascular injuries. Warning signs include the development of a progressive pulsatile mass, unexplained edema, and the absence of a palpable distal pulse. Differential diagnosis should include abscesses, hematomas, and neoplasms. Complications can range from severe pain, distal arterial embolism, and rupture and hemorrhage of the pseudoaneurysm to ulcer formation, which could culminate in amputation.^2,4^

Doppler ultrasonography has become the preferred imaging modality for the initial evaluation and diagnosis of pseudoaneurysms in this body region due to its high sensitivity and specificity, which range from 90-100% and 99-100%, respectively. The hallmark of pseudoaneurysms is an extraluminal blood flow pattern with variable echogenicity, a “yin-yang” mosaic sign, and turbulent systolic and diastolic blood flow, resulting in a “to-and-fro” Doppler waveform.^1,2,6,7^

## PART I – CLINICAL SITUATION

We present the case of a 25-year-old female patient with leucoderma who had a perforation injury to her left lower limb from a piece of glass. The injury resulted in severe bleeding immediately after the glass was removed. In response to the emergency, an improvised tourniquet was applied at home, and the patient was taken to the hospital.

During initial care at the emergency room, no signs of active bleeding were observed, so a simple suture was made at the site of the injury and, after the procedure, the patient was discharged with a prescription for anti-inflammatory medication.

Two weeks after the initial injury, the skin lesion was healing satisfactorily and the suture was removed. However, pain and edema persisted in the left lower limb, which led to suspicion of deep vein thrombosis, and venous Doppler ultrasound of the limb was requested.

**Figure 1:**
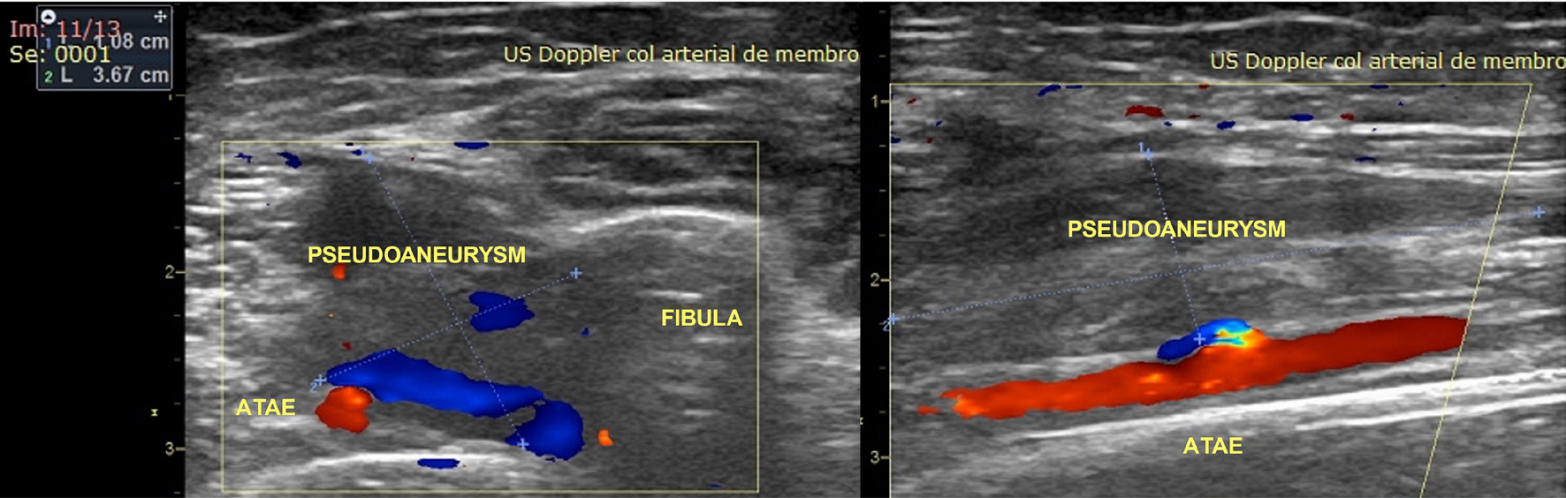
Color Doppler ultrasonography showing pseudoaneurysm of the anterior tibial artery. Left: transverse view showing a 1.08 cm × 3.67 cm pseudoaneurysm of the left anterior tibial artery (*ATAE* in the image). Right: longitudinal view showing the pseudoaneurysm and the solution of continuity in the anterior wall of the left anterior tibial artery, with aliasing at the site.

**Figure 2:**
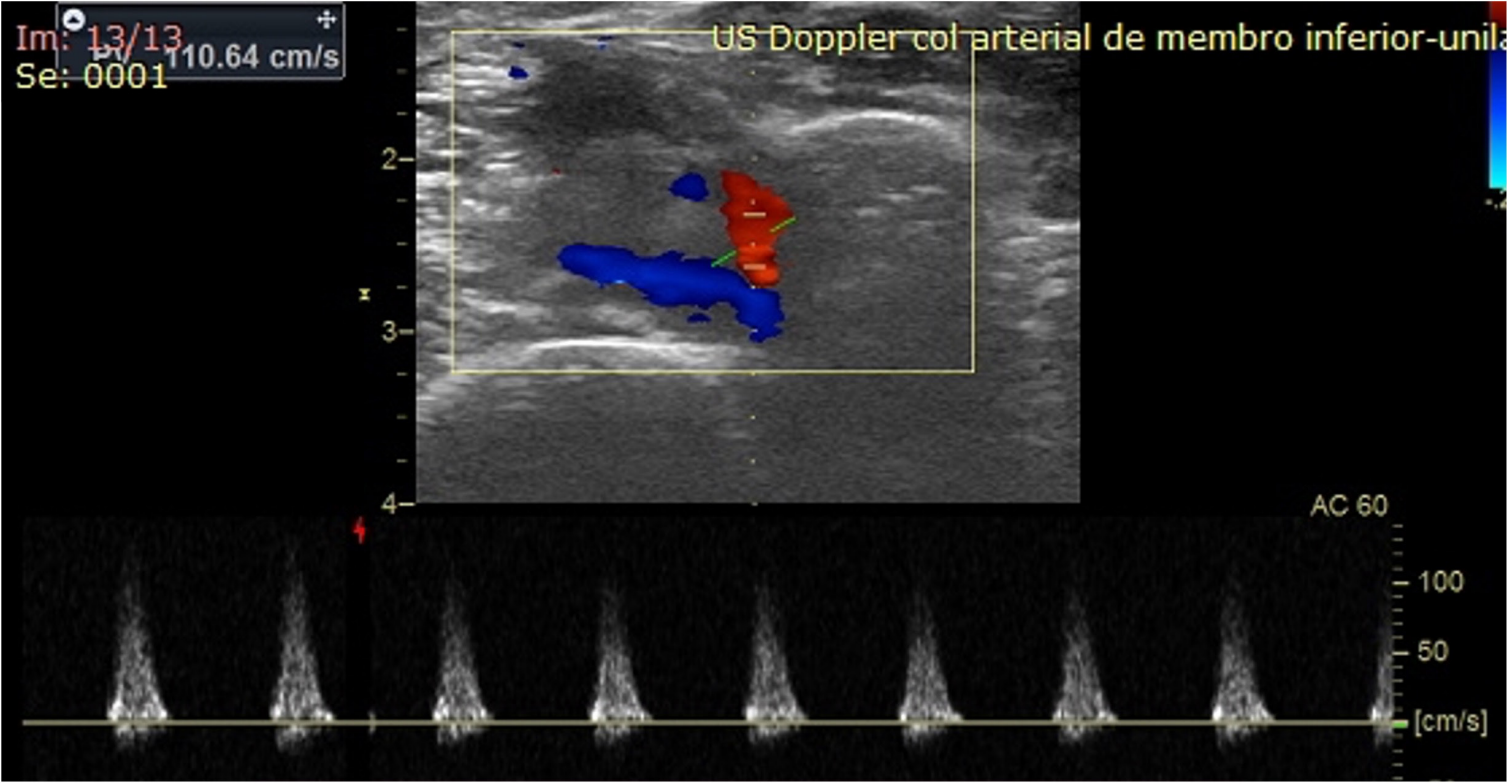
Color Doppler ultrasound showing high-resistance pulsatile flow within the pseudoaneurysm of the anterior tibial artery.

Deep vein thrombosis was ruled out by Doppler ultrasound, which was performed by a vascular surgeon. However, the analysis revealed heterogeneous echogenicity in the anterior wall of the middle third of the ATA, with dimensions of 1.83 cm × 1.56 cm in the anteroposterior and latero-lateral diameters, respectively (Figure 1). Color Doppler imaging showed high-resistance pulsatile flow within the pseudoaneurysm (Figure 2). It is important to emphasize that the distal segment of the anterior tibial artery and the other infragenicular arteries remained patent, exhibiting normal flow patterns.

## PART II – TREATMENT

Conventional surgery was the selected course of action. After administration of antibiotic prophylaxis with first-generation cephalosporin, the patient underwent spinal anesthesia with sedation. The precise site of the arterial injury was marked on the skin with the aid of Doppler ultrasound. A longitudinal incision was made along a predetermined path in the middle third of the leg. After proximal and distal control of the ATA and ligation of one of the anterior tibial veins, the site of the pseudoaneurysm was approached. The local hematoma was drained and, when a penetrating injury to the ATA was identified, a primary suture was made with 6-0 polypropylene monofilament.

**Figure 3:**
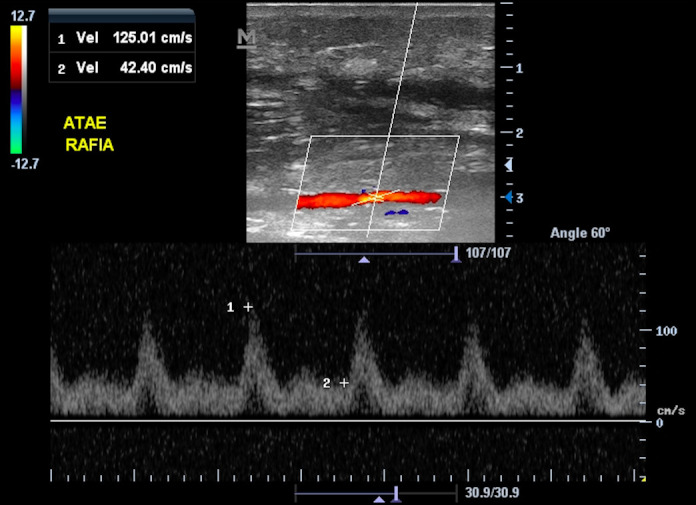
Color Doppler ultrasound performed 3 months after surgery, showing preserved flow in the left anterior tibial artery at the suture site.

On the first postoperative day, the patient’s general condition was good and she was discharged from hospital, with symptomatic medications and physical therapy prescribed. The patient was reassessed 3 months after discharge, presenting cutaneous hypoesthesia at the surgical site, while ultrasound showed that the ATA was patent (Figure 3).

## DISCUSSION

Arterial pseudoaneurysms resulting from lower limb trauma are rarely addressed in the literature because they tend to be asymptomatic or present with subtle clinical symptoms in late stages. However, the need for early diagnosis is imperative, given their potentially catastrophic complications.^8^

Penetrating injuries to the vessels are often severe, presenting bleeding, expanding hematoma, pulse deficit, or bruit, which allow immediate diagnosis. A lack of such signs often results in delayed diagnosis of vascular complications.^9^ In the present case, despite severe bleeding at the time of the injury, the initial emergency room evaluation did not reveal other significant signs or symptoms, which impeded early diagnosis.

It is important to note that, after 2 weeks, the patient was referred for Doppler ultrasound due to pain and edema in the limb, with deep vein thrombosis initially suspected. However, additional analysis of the arterial system revealed that it was a pseudoaneurysm of the ATA. Differential diagnoses of a pseudoaneurysm include deep vein thrombosis, hematoma, arteriovenous fistula, and soft tissue or bone tumors. The importance of imaging tests in such cases should be emphasized, not only to rule out other causes, but to accurately confirm a diagnosis of pseudoaneurysm.^2^

Persistent pain and swelling in the affected limb should raise suspicion of a pseudoaneurysm or arteriovenous fistula. These lesions, which are often progressive, can cause ongoing discomfort and compress adjacent nerves or tendons. It is critical for emergency physicians to promptly identify and initiate treatment for this potentially limb-threatening condition, which is easily achievable through point-of-care ultrasound, which has become an essential tool in emergency medicine.^6,9^

Therapeutic planning is usually based on the size of the vessel, the imaging characteristics, and the presence or absence of collateral flow in the region of the affected vessel, as well as the hemodynamic stability of the patient and the anatomy of the pseudoaneurysm.^1-3^

Standard pseudoaneurysm treatment includes surgical interventions such as arterial ligation, direct suture, and vein grafting. However, significant challenges to adequate vascular control can occur in such approaches, especially in complex situations involving extensive dissection and tissue distortion due to the pseudoaneurysm.^5,10^

In the present case, considering the patency of the other infrapatellar arteries, surgical ligation could have been performed. However, due to the limited extent of the lesion, primary suturing was selected to preserve the vascular patency of the ATA.

Several therapeutic alternatives, conservative and interventional, surgical and endovascular, have been reported in the literature. These include ultrasound-guided compression, thrombin injection, coil embolization, and stent implantation.^5,10^ The ideal therapeutic approach depends on the specific characteristics of each case, requiring careful evaluation to ensure the effectiveness and safety of the treatment.

Zhou et al.^4^ point out that not all treatment types are equally suitable for infrapopliteal pseudoaneurysms, considering the reduced diameter of the arteries in this region and their deep location. Open surgery in this location is invasive, while ultrasound-guided compression and isolated thrombin injection may not achieve a complete seal. Recent advances and endovascular technologies have shown promise for treating post-traumatic arterial injuries and pseudoaneurysms.

According to a review of case reports, 41% of infrapopliteal pseudoaneurysms were treated with ligation, 24% with endovascular embolization, 10% with direct arterial repair, 10% with covered stent, 4% with venous interposition repair, 4% with ultrasound-guided thrombin injection, 4% resolved spontaneously, and 2% were treated with ultrasound-guided compression.^9^

## CONCLUSIONS

Pseudoaneurysm of the ATA, although rare, is a challenging clinical entity that requires early diagnosis and an effective therapeutic approach. Integrating clinical evaluation, advanced imaging tests, such as ultrasound, and a comprehensive understanding of therapeutic options are essential for the effective management of these cases. The diversity of therapeutic approaches reported in the literature reflects the complexity of decision-making for this condition, emphasizing the importance of a personalized approach based on the vascular anatomy, the extent of the lesion, and the patient’s clinical conditions.

This study was duly evaluated and approved by the research ethics committee of the involved institution (certificate 78273124.5.0000.5297; decision number 6.707.252).
