# Supplementary material for: Post-traumatic pseudoaneurysm of the anterior tibial artery: therapeutic challenge
Source: J Vasc Bras. 2025 Mar 14;24:e20240120. doi: 10.1590/1677-5449.202401202 (PMC11922325; doi:10.1590/1677-5449.202401202)
Supplement: Translation

# Translation: Pseudoaneurisma pós-traumático da artéria tibial anterior: desafio terapêutico

**DOI:** 10.1590/1677-5449.202401201

**Authors:** Poliana Mazuchini Belai, Zaira Cristina Barbosa Assis, Thiago Felipe de Moraes Vieira, Gislaine dos Santos Rodrigues Vieira, Henrique Alves de Almeida, Thiago Vaz Lopes, João da Silva, Vinicius Tadeu Ramos da Silva Grillo

**Affiliations:** 1 Centro Universitário São Lucas – UNISL-Afya, Porto Velho, RO, Brasil.; 2 Instituto Vascular e Endovascular de Rondônia, Porto Velho, RO, Brasil.

**Keywords:** pseudoaneurisma, artéria tibial anterior, lesões vasculares

## Abstract

Pseudoaneurismas arteriais traumáticos de apresentação subaguda que envolvem as artérias infrageniculares são raramente abordados na literatura, devido à sua tendência de serem assintomáticos ou manifestarem sintomas clinicamente sutis em estágios tardios. Descrevemos o caso de uma paciente de 25 anos, com lesão perfurante no membro inferior provocada por estilhaço de vidro, que, após 2 semanas, apresentou dor e edema no membro, levando à suspeita de trombose venosa profunda. A ultrassonografia com Doppler venoso descartou a hipótese de trombose, porém revelou uma imagem com ecogenicidade heterogênea na parede anterior do terço médio da artéria tibial anterior, com fluxo em seu interior, sugerindo pseudoaneurisma. Optou-se por tratamento cirúrgico convencional e, após controle proximal e distal da artéria, procedeu-se à drenagem do hematoma local e à arteriorrafia primária da lesão puntiforme. O pseudoaneurisma das artérias infrapatelares, embora raro, destaca-se como uma entidade clínica desafiadora que requer diagnóstico precoce.

## INTRODUÇÃO

O pseudoaneurisma pode se originar em qualquer artéria do corpo humano. No membro inferior, a artéria poplítea é frequentemente afetada, seguida pela artéria femoral superficial e, subsequentemente, pela artéria tibial anterior (ATA). A ATA é o vaso sanguíneo mais frequentemente associado a pseudoaneurismas infrapatelares, embora sua incidência seja relativamente rara^1-3^ .

Pseudoaneurismas da ATA são eventos incomuns, que surgem devido a diversas influências iatrogênicas, traumas contusos ou penetrantes. Apesar de sua raridade, essas lesões estão associadas a uma morbidade significativa, muitas vezes representando uma ameaça para a integridade dos membros^1,2,4,5^ .

O intervalo de tempo decorrido entre a lesão inicial e o surgimento do pseudoaneurisma pode variar de horas a anos, dependendo da localização, do tamanho e das manifestações clínicas. Um elevado índice de suspeição clínica é essencial para o diagnóstico preciso de lesões vasculares. Sinais de alerta incluem o desenvolvimento de uma massa pulsátil progressiva, edema inexplicável e ausência de pulso distal palpável. O diagnóstico diferencial deve incluir abscessos, hematomas e neoplasias. As complicações podem variar desde dor intensa, embolia arterial distal, ruptura do pseudoaneurisma e hemorragia até a formação de úlceras, podendo culminar em necessidade de amputação^2,4^ .

A ultrassonografia (US) com Doppler emerge como a modalidade de imagem de eleição para avaliação e diagnóstico inicial de pseudoaneurismas nessa localização, devido à sua elevada sensibilidade e especificidade, variando de 90-100% e 99-100%, respectivamente. A característica distintiva do pseudoaneurisma é a exibição de um padrão de fluxo sanguíneo extraluminal, apresentando ecogenicidade variável, sinal colorido em mosaico (padrão “*yin yang*”) e fluxo turbulento tanto sistólico quanto diastólico, evidenciando a tradicional forma de onda “*to-and-fro*” (para frente e para trás)^1,2,6,7^ .

## PARTE I – SITUAÇÃO CLÍNICA

Apresentamos o caso de uma paciente do sexo feminino, 25 anos, leucoderma, com antecedente de lesão perfurante em membro inferior esquerdo provocada por estilhaço de vidro. A lesão resultou em sangramento acentuado imediatamente após a retirada do agente causador. Em resposta à emergência, foi aplicado um torniquete improvisado no domicílio, e a paciente foi conduzida ao hospital.

No atendimento inicial na unidade de pronto-socorro, não foram observados sinais de sangramento ativo. Diante disso, foi realizada uma sutura simples no local da lesão e, após o procedimento, a paciente foi liberada com prescrição de medicação anti-inflamatória.

Duas semanas após a lesão inicial, a paciente evidenciou cicatrização da lesão cutânea subsequente à retirada dos pontos de sutura. No entanto, persistiam dor e edema no membro inferior esquerdo, o que levou à suspeita de trombose venosa profunda (TVP). Diante desse cenário, foi solicitada uma US com Doppler venoso do membro.

Durante a realização da US com Doppler pelo cirurgião vascular, a suspeita de TVP foi descartada. No entanto, a análise revelou uma imagem com ecogenicidade heterogênea na parede anterior do terço médio da ATA, apresentando dimensões de 1,83 cm × 1,56 cm nos diâmetros anteroposterior e laterolateral, respectivamente (Figura 1). O exame colorido demonstrou um fluxo no interior da imagem ao modo Doppler (Figura 2). É importante ressaltar que o segmento distal da artéria tibial anterior e as demais artérias infrageniculares mantinham-se pérvias, exibindo padrões de fluxo dentro dos parâmetros de normalidade.

**Figura 1:** Ultrassonografia com Doppler colorido evidenciando o pseudoaneurisma da artéria tibial anterior. À esquerda: visão transversal mostrando pseudoaneurisma de artéria tibial anterior esquerda (ATAE) de 1,08 cm × 3,67 cm. À direita: visão longitudinal mostrando o pseudoaneurisma e a solução de continuidade na parede anterior da ATAE, com *aliasing* no local.

**Figura 2:** Ultrassonografia com Doppler colorido evidenciando fluxo pulsátil de alta resistência no interior do pseudoaneurisma da artéria tibial anterior.

## PARTE II – O QUE FOI FEITO

Optou-se por realizar tratamento cirúrgico convencional. A paciente foi submetida a raquianestesia com sedação, após administração de antibioticoprofilaxia com cefalosporina de primeira geração. O local preciso da lesão arterial foi demarcado na pele com o auxílio da US com Doppler. Uma incisão longitudinal foi realizada no trajeto demarcado, localizado no terço médio da perna. Após o controle proximal e distal da ATA e a ligadura de uma das veias tibiais anteriores, procedeu-se à abordagem do sítio do pseudoaneurisma. Realizou-se drenagem do hematoma local e, diante da identificação de lesão arterial puntiforme, foi realizada uma rafia primária com fio polipropileno 6-0.

A paciente recebeu alta hospitalar no primeiro dia de pós-operatório, apresentando-se em bom estado geral, com prescrição de medicações sintomáticas e fisioterapia motora. Na reavaliação realizada após 3 meses de pós-operatório, a paciente apresentava hipoestesia cutânea no sítio cirúrgico, enquanto a US evidenciava a perviedade da ATA (Figura 3).

**Figura 3:** Ultrassonografia com Doppler colorido realizada após 3 meses do pós-operatório, evidenciando fluxo preservado na artéria tibial anterior esquerda (ATAE) no local da arteriorrafia.

## DISCUSSÃO

Pseudoaneurismas arteriais decorrentes de traumas em membros inferiores são raramente abordados na literatura, dada a sua propensão a serem assintomáticos ou apresentarem sintomas clínicos sutis em estágios tardios. A necessidade de um diagnóstico precoce é imperativa, dadas as complicações potencialmente catastróficas associadas^8^ .

Lesões vasculares penetrantes frequentemente manifestam sinais de gravidade, como sangramento, hematoma em expansão, déficit de pulso ou sopro, possibilitando um diagnóstico imediato. A ausência desses sinais muitas vezes resulta em atrasos no diagnóstico de complicações vasculares^9^ . No caso relatado, apesar de sangramento acentuado no momento do trauma, a avaliação inicial no pronto-socorro não revelou outros sinais ou sintomas significativos, o que dificultou o diagnóstico precoce.

É crucial destacar que, após um período de 2 semanas, a paciente foi encaminhada para realizar a US com Doppler devido ao quadro de dor e edema no membro, inicialmente com suspeita de TVP. Contudo, a análise adicional do sistema arterial revelou tratar-se de um pseudoaneurisma da ATA. Entre os diagnósticos diferenciais de um pseudoaneurisma, estão TVP, hematoma, fístula arteriovenosa e tumores de partes moles ou de origem óssea. Ressalta-se a importância do papel dos exames de imagem nesses casos, não apenas para descartar outras causas, mas também para confirmar de maneira precisa o diagnóstico de pseudoaneurisma^2^ .

A persistência de dor e edema no membro afetado deve suscitar a suspeita de um pseudoaneurisma ou formação de fístula arteriovenosa. Essas lesões, comumente de natureza progressiva, têm o potencial de causar desconforto contínuo, bem como compressão de nervos ou tendões adjacentes. É fundamental que os médicos de emergência possuam a capacidade de identificar prontamente e iniciar o tratamento para essa condição potencialmente ameaçadora dos membros. Essa abordagem pode ser facilmente alcançada por meio do uso de US no local de atendimento, que se estabeleceu como uma ferramenta essencial na prática da medicina de emergência^6,9^ .

O planejamento terapêutico costuma estar condicionado ao tamanho do vaso, às características de imagem e à presença ou ausência de fluxo colateral no território do vaso afetado, bem como à estabilidade hemodinâmica do paciente e à anatomia do pseudoaneurisma^1-3^ .

O tratamento padrão para o pseudoaneurisma compreende intervenções cirúrgicas, tais como ligadura arterial, sutura direta e enxerto venoso. Contudo, tais abordagens podem apresentar desafios significativos na obtenção de controle vascular adequado, especialmente em situações complexas em que ocorre dissecção extensa em um campo operatório com os planos distorcidos devido ao pseudoaneurisma^5,10^ .

No caso apresentado, uma opção terapêutica poderia ter sido a ligadura cirúrgica, considerando a perviedade das demais artérias infrapatelares. No entanto, devido à extensão limitada da lesão, a escolha recaiu sobre a arteriorrafia primária, visando preservar a perviedade vascular da ATA.

Diversas alternativas terapêuticas, tanto conservadoras quanto intervencionistas cirúrgicas e endovasculares, foram relatadas na literatura. Estas incluem compressão guiada por US, injeção de trombina, embolização com mola e implantação de endoprótese^5,10^ . A seleção da abordagem terapêutica ideal depende das características específicas de cada caso, demandando uma avaliação criteriosa para garantir a eficácia e a segurança do tratamento.

Zhou et al.^4^ destacam que nem todas as abordagens são igualmente adequadas para o tratamento de pseudoaneurismas infrapoplíteos, considerando o diâmetro reduzido das artérias nessa região e sua localização profunda. A cirurgia aberta nesse local é invasiva, enquanto a compressão guiada por US e a injeção isolada de trombina podem apresentar desafios para a obtenção de uma vedação completa. O avanço recente e a aplicação de tecnologias endovasculares têm se mostrado promissores no tratamento de lesões arteriais e pseudoaneurismas pós-traumáticos.

Uma revisão do manejo de casos relatados na literatura demonstra que 41% dos pseudoaneurismas infrapoplíteos foram tratados com ligadura, 24% com embolização endovascular, 10% com reparo arterial direto, 10% com stent coberto, 4% com reparo com interposição venosa, 4% com injeção ecoguiada de trombina, 4% resolveram espontaneamente e 2% foram tratados com compressão guiada por US^9^ .

## CONCLUSÃO

O pseudoaneurisma da ATA, embora raro, destaca-se como uma entidade clínica desafiadora, que requer diagnóstico precoce e abordagem terapêutica efetiva. A integração de avaliação clínica, exames de imagem avançados, como US, e compreensão abrangente das opções terapêuticas é fundamental para o manejo efetivo desses casos. A diversidade de abordagens terapêuticas relatadas na literatura reflete a complexidade da tomada de decisões diante dessa condição, enfatizando a importância de uma abordagem personalizada com base na anatomia vascular, extensão da lesão e condições clínicas do paciente.

Este estudo foi devidamente avaliado e aprovado por um Comitê de Ética em Pesquisa (CAAE 78273124.5.0000.5297 e parecer número 6.707.252).
